# Approvals and Timing of New Formulations of Novel Drugs Approved by the US Food and Drug Administration Between 1995 and 2010 and Followed Through 2021

**DOI:** 10.1001/jamahealthforum.2022.1096

**Published:** 2022-05-20

**Authors:** Ravi Gupta, Christopher J. Morten, Angela Y. Zhu, Reshma Ramachandran, Nilay D. Shah, Joseph S. Ross

**Affiliations:** 1 National Clinician Scholars Program, University of Pennsylvania, Philadelphia; 2 Corporal Michael J. Crescenz VA Medical Center, Philadelphia, Pennsylvania; 3 Columbia Law School, New York, New York; 4 Division of Biostatistics, Department of Biostatistics, Epidemiology and Informatics, University of Pennsylvania Perelman School of Medicine, Philadelphia; 5 National Clinician Scholars Program, Department of Internal Medicine, Yale School of Medicine, New Haven, Connecticut; 6 VA Connecticut Healthcare System, West Haven, Connecticut; 7 Delta Air Lines, Atlanta, Georgia; 8 Section of General Internal Medicine, Department of Medicine, Yale School of Medicine, New Haven, Connecticut; 9 Department of Health Policy and Management, Yale School of Public Health, New Haven, Connecticut; 10 Center for Outcomes Research and Evaluation, Yale New Haven Hospital, New Haven, Connecticut

## Abstract

**Importance:**

New formulations of prescription drugs can improve convenience and tolerability for patients, but they also constitute manufacturer strategies to extend brand-name drug market exclusivity periods.

**Objective:**

To examine whether new formulations of brand-name novel drugs were associated with novel drugs’ sales and/or therapeutic value, as well as characterize first new formulations’ approval timing relative to the novel drug’s generic approval.

**Design, Setting, and Participants:**

This cross-sectional study used the Drugs@FDA database to identify all novel tablet and capsule drugs approved by the US Food and Drug Administration (FDA) between 1995 and 2010 and followed through December 31, 2021.

**Exposures:**

Novel drugs’ blockbuster status, defined as annual sales of $1 billion or greater, and therapeutic value, measured by (1) accelerated approval status, (2) World Health Organization Model Lists of Essential Medicines inclusion, (3) innovativeness, and (4) clinical usefulness.

**Main Outcomes and Measures:**

Approval of a new formulation and timing relative to a novel drug’s first generic’s approval.

**Results:**

Among the 206 novel drugs in tablet or capsule form approved by the FDA from 1995 to 2010, 81 (39.3%) were followed by an FDA-approved new formulation, and 167 (81.1%) had a generic version as of December 31, 2021. In multivariable analyses, new formulations were statistically significantly more likely among blockbuster drugs vs not (58.2% vs 27.6%; adjusted odds ratio [AOR], 4.72; 95% CI, 2.26-9.87; *P* < .001) and those granted accelerated approval vs not (50.0% vs 37.6%; AOR, 5.48; 95% CI, 1.52-19.67; *P* = .009), and less likely among orphan products vs not (11.8% vs 44.8%; AOR, 0.13; 95% CI, 0.03-0.52; *P* = .004). Essential medicine listing vs no listing (47.8% vs 36.9%; AOR, 1.32; 95% CI, 0.52-3.34; *P* = .56), first-in-class or advance-in-class status vs addition-to-class status (37.8% vs 40.5%; AOR, 0.71; 95% CI, 0.32-1.58; *P* = .40), and categorization as clinically useful vs not useful (40.9% vs 44.8%; AOR, 0.81; 95% CI, 0.34-1.92; *P* = .64) were not associated with increased likelihood of a new formulation. First new formulations were statistically significantly less likely to be approved after the novel drug’s first generic approval (84.6% vs 15.4%; *P* < .001).

**Conclusions and Relevance:**

In this cross-sectional study of novel drugs in tablet or capsule form approved by the FDA between 1995 and 2010, manufacturers pursued new formulations of best-selling brand-name drugs and those granted accelerated approval but did so less frequently once generic competitors entered the market. Other measures of therapeutic value were not associated with new formulations.

## Key Points

**Questions:**

Are sales or therapeutic value associated with approval of new formulations of brand-name novel drugs, and does timing coincide with generic competition?

**Findings:**

In this cross-sectional study of 206 brand-name drugs approved in tablet or capsule form by the US Food and Drug Administration between 1995 and 2010, approval of new formulations was 4 times more likely among blockbuster drugs and 5.5 times more likely among drugs granted accelerated approval. First new formulation approval was statistically significantly lower after approval of generic competition.

**Meaning:**

Manufacturers pursue new formulations of best-selling brand-name drugs and those granted accelerated approval but less frequently once generic competition begins.

## Teaser

This cross-sectional study examines whether new formulations of brand-name novel drugs were associated with novel drugs’ sales and/or therapeutic value as well as characterizes new formulations’ approval timing relative to the novel drug’s generic approval.

## Introduction

Brand-name drug manufacturers can modify existing drugs through various means, including changes in delivery mechanisms to decrease dosing frequency, slight chemical alterations, and combinations of multiple active ingredients.^1^ Such new formulations can be useful for patients by increasing convenience, improving adherence through dosing frequency reductions or better tolerability, and offering additional treatments for diseases.^2,3,4,5^ However, in some cases new formulations, particularly tablets and capsules, may not be clinically superior to the novel drug,^6,7,8^ and their potential convenience benefits may be outweighed by their cost. Moreover, whether new formulations are more common for drugs that are therapeutically valuable remains unclear.

Another concern is that new formulations constitute one of numerous well-described strategies—called “evergreening” or “life-cycle management”—used by brand-name drug manufacturers to extend periods of market exclusivity protection and thus revenue associated with drugs,^1,9,10,11,12^ particularly as they face generic competition.^13^ One report found that new formulation approvals are considerably higher around the first generic’s entry and that manufacturers decide when to pursue new formulations based on the extent to which their sales would cut into those of the novel drug.^14^ Strategically delaying new formulation applications until the time of generic entry complicates the ability of generic versions to achieve widespread use and lower prescription drug spending. In a strategy known as “product hopping,”^9,15^ for example, brand-name manufacturers may simultaneously heavily promote the new formulation over the novel drug and rely on laws that prevent pharmacies from automatically substituting the new formulation with generic versions of the novel drug. Product-hopping strategies lead to substantial excess spending owing to the delayed availability of potentially useful formulations^16,17,18^ and the introduction of extended-release formulations^8,19^ and fixed-dose combination drugs^20,21^ of uncertain marginal value.

Previous studies have characterized specific examples of product hopping,^16,17,22,23^ assessed new formulations over narrow time frames^24^ or formulation types,^19,25^ or sought to determine the therapeutic value of new formulations.^6,26,27,28^ To our knowledge, no prior studies, however, have examined additional characteristics, including therapeutic value, of novel drugs for which manufacturers pursued new formulations. Studying the therapeutic value of novel drugs that are formulated again holds particular salience for prescription drug pricing reform and incentives for pharmaceutical innovation. In this cross-sectional study, we assessed the association between the presence of a new formulation and the novel drug’s sales and therapeutic value using 4 different, objective measures. We examined the timing of the first new formulation’s approval since the novel drug’s approval and before and after the first generic version’s approval.

## Methods

### Data and Study Sample

We used the Drugs@FDA database to identify all new drug applications for new molecular entities (novel drugs) in tablet and capsule form approved by the US Food and Drug Administration (FDA) between January 1, 1995, and December 31, 2010, excluding tentative approvals, biological treatments, over-the-counter drugs, and duplicate listings (eFigure 1 in the Supplement). We excluded novel drugs withdrawn from the market due to safety concerns. For the secondary outcome of new formulation approval timing, we excluded drugs with active patent protections, other market exclusivity, or ongoing litigation as of December 2021, identified from the FDA’s Orange Book drug database, public sources, company press releases, and US Securities and Exchange Commission documents, because these drugs were not yet likely to face generic competition.

For each novel drug in the final cohort, new formulations were identified as of December 31, 2021, using Drugs@FDA. New formulations were included if they were^19^ (1) approved through a new drug application with at least 1 active ingredient matching the novel drug’s, (2) approved as a tablet or capsule, (3) approved more than 6 months after the novel drug (to account for possible regulatory delays in simultaneous application filings), and (4) included in an application filed by the same manufacturer as the novel drug or a manufacturer that had acquired or merged with the original manufacturer or a different manufacturer but with the drug sharing the source brand name. We excluded new formulations that were new active ingredients, marketed but unapproved, or only divisible dosage changes (eg, change to 40-mg dose from an existing 20-mg dose). For fixed-dose combination drugs, these criteria were applied to each active ingredient of the combination individually to be included as a new formulation, if the active ingredient met the inclusion criteria for novel drugs. For example, amlodipine besylate/atorvastatin calcium (Caduet) was included as a new combination formulation for atorvastatin calcium (Lipitor) but not amlodipine besylate (Norvasc) because amlodipine besylate was first approved in 1992, before the start of the study period.

For each novel drug, we then used Drugs@FDA to identify the first FDA-approved bioequivalent generic drug by matching active ingredient and formulation as of December 31, 2021. We disregarded multiple dosage strengths approved on the same date and counted the generic drug only once. We excluded tentative approvals and authorized generics.^29^

This study followed the Strengthening the Reporting of Observational Studies in Epidemiology (STROBE) reporting guideline for cross-sectional studies. Institutional review board approval was not necessary because this study did not include human participant data.

### Sales

Drugs with blockbuster status have annual global sales of $1 billion or greater, indicating large profits.^30^ Using annual sales extracted from public sources, company press releases, and US Securities and Exchange Commission documents, each novel drug in the present cohort was classified by whether it was a blockbuster drug based on whether it had, during any year since its approval, annual global sales of $1 billion or greater.

### Therapeutic Value

The therapeutic value of a drug to patients is dependent on a number of factors, including whether the drug is a promising treatment beyond existing therapeutic alternatives, clinically important and indicated for treatment of a widely prevalent disease, an innovative advance, and clinically useful. We estimated the therapeutic value of each novel drug in the sample using these 4 proxy measures.^31^

The promise of a novel drug was assessed by whether it was granted accelerated approval by the FDA. Drugs that received accelerated approval were identified using Drugs@FDA, including FDA approval letters. Therapeutics that address a serious unmet medical need may be eligible for accelerated approval status, allowing the FDA to approve the drug on the basis of surrogate markers of disease as clinical trial end points.^32^

Clinical importance of novel drugs was assessed through a proxy measure of whether the drug was included in the World Health Organization’s (WHO) Model Lists of Essential Medicines,^33^ which list drugs that address a population’s priority health care needs. These lists offer a straightforward metric of both the drug’s utility and the relative prevalence of the disease(s) for which it is indicated.

To assess whether each novel drug was considered innovative, we followed a schematic established by FDA research,^34^ which categorized drugs as first in class, advance in class, or addition to class. Using this schematic, innovative drugs that were the first approved within their respective drug class were considered first in class because they represent a new pathway for treating a disease. Drugs that were not first in class but received priority review status were categorized as advance in class. All other drugs were categorized as addition to class. In the present analysis, we grouped first in class and advance in class together compared with addition to class.

We assessed clinical usefulness of each novel drug by using an established source^31,35^ of ratings by the French drug industry watchdog Prescrire International,^36^ an independent, nonprofit organization that reviews new treatments. Drugs that were categorized by Prescrire as “possibly helpful,” “offers an advantage,” “a real advance,” or “bravo” were grouped together as “clinically useful.” Drugs that were categorized as “nothing new” and “not acceptable” were grouped together as “clinically not useful.” The remaining drugs were categorized as “judgment reserved.” For drugs for which Prescrire ratings were not available, we reviewed Prescrire materials and guidelines to extrapolate assessments. If no statements could be found, drugs were grouped together with judgment reserved.

### Other Drug Characteristics

Drugs are granted orphan status by the FDA if they treat a rare disease, defined as affecting fewer than 200 000 individuals in the US annually. The Orphan Drug Product designation database was used to determine whether novel drugs had received designation as orphan products for the first approval indication.^37,38^

Using the WHO’s Anatomical Therapeutic Classification system,^39^ we categorized each novel drug into 1 of 9 treatment classes based on the indication for which the drug was first approved: autoimmune or musculoskeletal; cancer; cardiovascular, diabetes, or hyperlipidemia; gastrointestinal; genitourinary/sex hormones; infectious disease; neurology; psychiatry; and other (eTable 1 in the Supplement). Finally, we categorized the approval year for each novel drug into 3-year increments from 1995 to 2010.

### Presence and Timing of New Formulations

The primary outcome was whether each novel drug in the sample was followed by at least 1 new FDA-approved formulation of the same drug. The secondary outcome was the timing of the first new formulation approval, categorized into before and after the first generic approval. The timing of the first new formulation approval, particularly relative to the novel drug’s first generic competitor’s approval, is an important indicator of whether the new formulation is a potential tool for evergreening.

### Statistical Analysis

We used descriptive statistics to characterize novel drugs and their first new formulations and first generics. We conducted bivariate and multivariable logistic regression analyses with presence of at least 1 new formulation as the outcome, and we report unadjusted odds ratios (ORs) and adjusted ORs (AORs), respectively, with 95% CIs for 8 novel drug characteristics: (1) blockbuster status, (2) accelerated approval status, (3) WHO Model Lists of Essential Medicines inclusion, (4) orphan product, (5) innovation status, (6) clinical usefulness (based on Prescrire ratings), (7) therapeutic area, and (8) approval year.

For novel drugs with at least 1 new formulation and 1 generic, we produced Kaplan-Meier estimates to plot the presence of a new formulation as a function of time since the novel drug’s approval, with 2-sided log-rank tests to assess for differences in events over time among all 8 novel drug characteristics. We then performed a χ^2^ goodness-of-fit test to examine whether the observed proportion of new formulations before and after the first generic approval was statistically significantly different from the expected equal proportions. An additional timing analysis was specified to account for regulatory approval delays by examining the likelihood of a new formulation in different windows relative to the novel drug’s first generic approval.

All statistical tests were 2-tailed and used *P* = .05 as a threshold for statistical significance. We used Stata, version 16 (StataCorp), and R, version 4.0.4 (R Foundation for Statistical Computing), to conduct all analyses.

### Additional Analyses

Given the possibility of blockbuster drugs also being most therapeutically valuable, we conducted bivariate and multivariable logistic regression analyses with blockbuster status as the outcome and each measure of therapeutic value and other drug characteristics as covariates. In addition, because of likely correlation between different measures of therapeutic value, we performed bivariate analyses using χ^2^ tests to examine associations between measures.

## Results

### Characteristics of Novel Drugs

**Figure 1.**
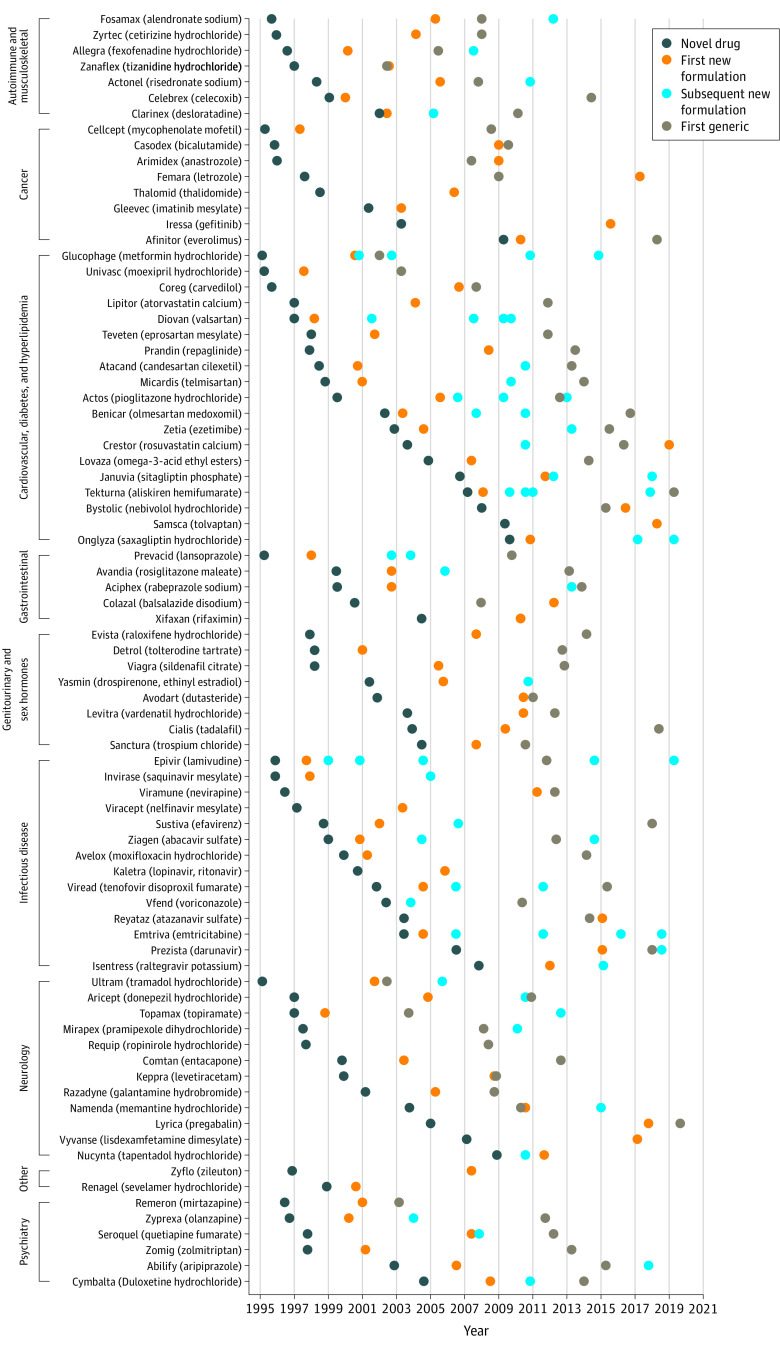
Timeline of Novel Tablets and Capsules Approved by the US Food and Drug Administration, 1995-2010, Followed by New Formulation and Generic Version Approvals Grouped by Therapeutic Area (n = 81)

A total of 206 novel drugs in tablet or capsule formulation were approved by the FDA between 1995 and 2010 (Table 1). Eighty-one (39.3%) were followed by at least 1 new FDA-approved formulation (Figure 1 and eFigure 2 in the Supplement), and 167 (81.1%) had at least 1 generic version approved by the FDA as of December 31, 2021 (Table 2). Among the 206 novel drugs, nearly one-third (n = 65 [31.6%]) were followed by at least 1 new formulation and a generic.

**Table 1.** Characteristics of Novel Drugs in Tablet or Capsule Formulation Approved by the US Food and Drug Administration, 1995-2010 (N = 206)

Characteristic	No. (%)
Blockbuster status^a^	
Yes	79 (38.4)
No	127 (61.7)
Accelerated approval	
Yes	28 (13.6)
No	178 (86.4)
WHO essential medicine	
Yes	46 (22.3)
No	160 (77.7)
Innovation status	
First in class or advance in class	90 (43.7)
Addition to class	116 (56.3)
Clinical usefulness^b^	
Judgment reserved	44 (21.4)
Clinically not useful	96 (46.6)
Clinically useful	66 (32.0)
Orphan status	
Yes	34 (16.5)
No	172 (83.5)
Therapeutic area	
Autoimmune or musculoskeletal	14 (6.8)
Cancer	25 (12.1)
Cardiovascular, diabetes, or hyperlipidemia	39 (18.9)
Gastrointestinal	15 (7.3)
Genitourinary/sex hormones	18 (8.7)
Infectious disease	36 (17.5)
Neurology	33 (16.0)
Psychiatry	17 (8.3)
Other	9 (4.4)
Approval year	
1995-1997	58 (28.2)
1998-2000	48 (23.3)
2001-2003	31 (15.1)
2004-2006	31 (15.1)
2007-2010	38 (18.5)

Abbreviation: WHO, World Health Organization.

^a^ Drugs were categorized as blockbuster if they had annual global sales of $1 billion during any year after the novel drug’s approval.

^b^ Clinical usefulness was determined through review of ratings from Prescrire International, the French drug industry watchdog. If drugs were not evaluated by Prescrire, they were categorized as “judgment reserved.” If the drugs were categorized by Prescrire as “possibly helpful,” “offers an advantage,” “a real advance,” or “bravo,” they were grouped together as “clinically useful.” If the drugs were categorized by Prescrire as “nothing new” or “not acceptable,” they were grouped together as “clinically not useful.”

**Table 2.** Characteristics and Approval Timing of New Formulations of Novel Tablet or Capsule Drugs Approved by the US Food and Drug Administration, 1995-2010 (N = 206)

Characteristics and timing of new formulations and generics	No. (%)
No. of new formulations per novel drug	
0	125 (60.7)
1	45 (21.8)
2	23 (11.2)
≥3	13 (6.3)
New formulations and generics of novel drugs	
With ≥1 new formulation	81 (39.3)
With ≥1 generic	167 (81.1)
With 0 new formulations and 0 generics	23 (11.2)
With ≥1 new formulation and 1 generic	65 (31.6)
First new formulation type (n = 81)	
Dosage form^a^	34 (41.9)
Combination	29 (35.8)
Indication	13 (16.0)
Other	5 (6.2)
Approval timing of novel drug, new formulation, and generic, median (IQR), y	
Novel drug and first new formulation approval	4.6 (2.3-8.6)
Novel drug and first generic approval	11.4 (8.1-14.5)
First new formulation approval and first generic approval	5.9 (1.5-10.9)

^a^ Carvedilol had a new formulation that was both a switch from tablet to extended-release capsule (ie, a new dosage form) and approved as carvedilol phosphate (ie, a new active ingredient). This drug was included in the sample as a new dosage form.

Among these 206 drugs, the most common clinical indications were for cardiovascular disease, diabetes, or hyperlipidemia (n = 39 [18.9%]), followed by infectious disease (n = 36 [17.5%]) and neurologic conditions (n = 33 [16.0%]). One-sixth of novel drugs were designated as orphan products (n = 34 [16.5%]). More than one-third were blockbuster drugs (n = 79 [38.4%]). Overall, 121 (58.7%) drugs qualified for at least 1 proxy of therapeutic value (eTable 2 in the Supplement). Less than one-fifth were granted accelerated approval by the FDA (n = 28 [13.6%]). One-fifth were considered essential medicines by WHO (n = 46 [22.3%]). Nearly half were considered innovative, categorized as first-in-class or advance-in-class drugs (n = 90 [43.7%]). Approximately one-third were considered clinically useful based on Prescrire ratings (n = 66 [32.0%]), whereas nearly half were considered clinically not useful (n = 96 [46.6%]).

### Presence and Timing of New Formulations and Generics

Approximately one-fifth of novel drugs had 1 new FDA-approved formulation (n = 45 [21.8%]), 23 (11.2%) had 2, and 13 (6.3%) had 3 or more (Table 2). Among the 81 drugs with at least 1 new formulation, nearly half of the first new formulations were new dosage forms (eg, extended-release versions) (n = 34 [41.9%]), more than one-third were combination drugs (n = 29 [35.8%]), and nearly one-sixth were approved for new indications (n = 13 [16.0%]). Among drugs with at least 1 new formulation, the median (IQR) time from novel drug to first new formulation approval was 4.6 (2.3-8.6) years. Among drugs with at least 1 generic, the median (IQR) time from novel drug to first generic approval was 11.4 (8.1-14.5) years. For novel drugs that had both a new formulation and a generic, the first new formulation was approved a median (IQR) of 5.9 (1.5-10.9) years prior to the first generic’s approval.

### Factors Associated With Presence of a New Formulation

In multivariable analyses, new formulations were statistically significantly more likely among blockbuster drugs (AOR, 4.72; 95% CI, 2.26-9.87; *P* < .001) and novel drugs granted accelerated approval (AOR, 5.48; 95% CI, 1.52-19.67; *P* = .009), and less likely among orphan products (AOR, 0.13; 95% CI, 0.03-0.52; *P* = .004) (Table 3). Essential medicine list inclusion (AOR, 1.32; 95% CI, 0.52-3.34; *P* = .56), first-in-class or advance-in-class status (AOR, 0.71; 95% CI, 0.32-1.58; *P* = .40), and categorization as clinically useful based on Prescrire ratings (AOR, 0.81; 95% CI, 0.34-1.92; *P* = .64) were not associated with increased likelihood of a new formulation. There were no statistically significant differences in the likelihood of new formulations by therapeutic area.

**Table 3.** New Formulation Approval and Associations Between Approval and Characteristics of Novel Tablets and Capsules Approved by the US Food and Drug Administration, 1995-2010 (N = 206)

Novel drug characteristic	Proportion with a new formulation, % (95% CI)	Bivariate analysis		Multivariable analysis	
		Odds ratio (95% CI)	*P* value	Adjusted odds ratio (95% CI)	*P* value
Blockbuster status					
No	27.6 (20.5-36.0)	1 [Reference]	NA	1 [Reference]	NA
Yes	58.2 (47.1-68.6)	3.66 (2.03-6.63)	<.001	4.72 (2.26-9.87)	<.001
Accelerated approval					
No	37.6 (30.8-45.0)	1 [Reference]	NA	1 [Reference]	NA
Yes	50.0 (32.2-67.8)	1.66 (0.74-3.69)	.22	5.48 (1.52-19.67)	.009
WHO essential medicine					
No	36.9 (29.7-44.7)	1 [Reference]	NA	1 [Reference]	NA
Yes	47.8 (33.9-62.1)	1.57 (0.81-3.04)	.18	1.32 (0.52-3.34)	.56
Orphan status					
No	44.8 (37.5-52.3)	1 [Reference]	NA	1 [Reference]	NA
Yes	11.8 (4.5-27.6)	0.16 (0.06-0.49)	.001	0.13 (0.03-0.52)	.004
Innovation status					
Addition to class	40.5 (31.9-49.7)	1 [Reference]	NA	1 [Reference]	NA
First in class or advance in class	37.8 (28.3-48.2)	0.89 (0.51-1.57)	.69	0.71 (0.32-1.58)	.40
Clinical usefulness^a^					
Clinically not useful	44.8 (35.1-54.9)	1 [Reference]	NA	1 [Reference]	
Judgment reserved	25.0 (14.4-39.8)	0.41 (0.19-0.91)	.03	0.56 (0.21-1.51)	.25
Clinically useful	40.9 (29.7-53.1)	0.85 (0.45-1.61)	.62	0.81 (0.34-1.92)	.64
Therapeutic area					
Autoimmune or musculoskeletal	50.0 (25.8-74.2)	1 [Reference]	NA	1 [Reference]	NA
Cancer	32.0 (16.8-52.3)	0.47 (0.12-1.80)	.27	0.78 (0.14-4.55)	.79
Cardiovascular, diabetes, or hyperlipidemia	48.7 (33.6-64.1)	0.95 (0.28-3.22)	.93	1.46 (0.36-5.90)	.59
Gastrointestinal	33.3 (14.5-59.5)	0.50 (0.11-2.24)	.37	1.56 (0.26-9.20)	.63
Genitourinary/sex hormones	44.4 (23.9-67.1)	0.80 (0.20-3.25)	.76	1.68 (0.33-8.48)	.53
Infectious disease	38.9 (24.5-55.5)	0.64 (0.18-2.21)	.48	0.72 (0.15-3.54)	.68
Neurology	36.4 (21.9-53.8)	0.57 (0.16-2.02)	.39	1.52 (0.36-6.42)	.57
Psychiatry	35.3 (16.7-59.7)	0.55 (0.13-2.31)	.41	0.81 (0.15-4.30)	.80
Other	22.2 (5.6-58.1)	0.29 (0.04-1.89)	.19	1.55 (0.18-13.33)	.69
Approval year					
1995-1997	53.4 (40.6-65.9)	1 [Reference]	NA	1 [Reference]	NA
1998-2000	37.5 (25.0-51.9)	0.52 (0.24-1.14)	.10	0.37 (0.14-0.94)	.04
2001-2003	54.8 (37.3-71.2)	1.06 (0.44-2.54)	.90	0.98 (0.35-2.76)	.98
2004-2006	22.6 (11.1-40.5)	0.25 (0.09-0.68)	.007	0.19 (0.06-0.59)	.004
2007-2010	21.1 (10.8-36.9)	0.23 (0.09-0.59)	.002	0.23 (0.08-0.68)	.008

Abbreviations: NA, not applicable; WHO, World Health Organization.

^a^ Clinical usefulness was determined through review of ratings from Prescrire International, the French drug industry watchdog. If drugs were not evaluated by Prescrire, they were categorized as “judgment reserved.” If the drugs were categorized by Prescrire as “possibly helpful,” “offers an advantage,” “a real advance,” or “bravo,” they were grouped together as “clinically useful.” If the drugs were categorized by Prescrire as “nothing new” or “not acceptable,” they were grouped together as “clinically not useful.”

### Timing of New Formulations Relative to Generics

**Figure 2.**
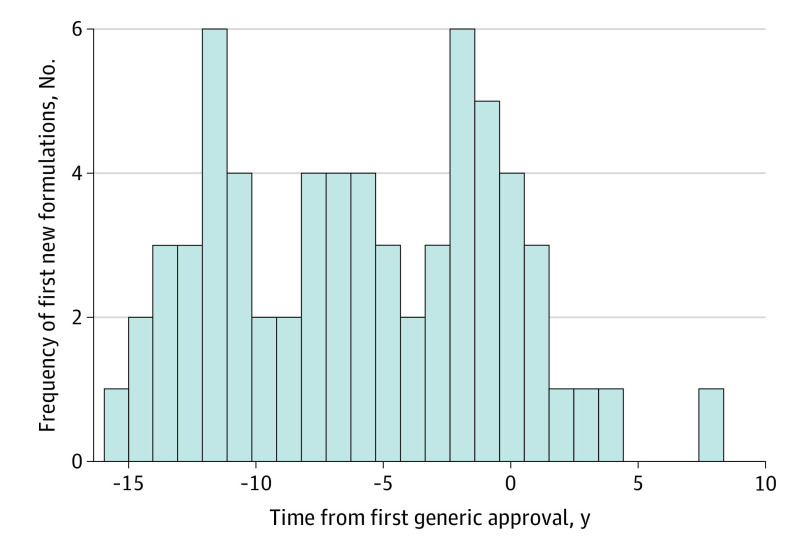
Timing of Novel Tablet and Capsule First New Formulation Approved by the FDA Relative to Generic Drug Approval (n = 65) FDA indicates US Food and Drug Administration.

Among the 65 novel drugs with at least 1 new formulation and a generic, 55 (84.6%) new formulations were approved before the novel drug’s first generic approval and 10 (15.4%) were approved after generic approval (Figure 2). First new formulations were less likely to be approved after the novel drug’s first generic approval compared with before generic approval (χ^2^ = 31.2; *P* < .001). Results from the additional timing analysis in different time windows relative to the first generic approval are reported in eMethods in the Supplement.

### Additional Analyses

In multivariable analysis, drugs considered essential medicines were more likely to be blockbuster drugs (AOR, 4.94; 95% CI, 1.91-12.76; *P* = .001). Blockbuster status was not associated with accelerated approval status (AOR, 0.82; 95% CI, 0.24-2.80; *P* = .76), first-in-class or advance-in-class status (AOR, 1.37; 95% CI, 0.67-2.82; *P* = .39), or categorization as clinically useful based on Prescrire ratings (AOR, 0.93; 95% CI, 0.42-2.06; *P* = .86). Results from bivariate analyses were similar (eTable 3 in the Supplement). Measures of therapeutic value were statistically significantly correlated with each other (eTable 4 in the Supplement).

## Discussion

In this analysis of 206 novel drugs approved in tablet or capsule form by the FDA from 1995 to 2010, one-third had at least 1 new formulation approved through December 2021, with blockbuster drugs 4 times more likely and drugs granted accelerated approval 5.5 times more likely to have a new formulation. Other measures of therapeutic value, however, were not associated with an increased likelihood of a new formulation. Additionally, there was a sharp decrease in new formulation approvals after the first generic’s approval. Taken together, these results suggest that revenue is a substantial driver of whether and when a manufacturer secures FDA approval of the first new formulation of existing drugs, reinforcing concerns that manufacturers are using evergreening strategies to maintain revenue and avoid generic competition.

These findings are supported by a recent report, which found that top-selling novel drugs were twice as likely to be followed by a new formulation.^14^ The present study advances this literature by also examining measures of novel drugs’ therapeutic value to determine whether products of greatest importance to patients are being formulated again to improve convenience or tolerability. With the exception of accelerated approval status, no measure of therapeutic value, including innovativeness and Prescrire rating, was associated with approval of a new formulation. This may, in part, reflect the considerable number of novel drugs in this study that are similar to existing drugs within a therapeutic class. Furthermore, because drugs that are granted accelerated approval are most commonly indicated to treat cancer,^40^ it is perhaps unsurprising that these drugs were more likely to be formulated again to improve patient convenience or tolerability.

The analyses of new formulations’ approval timing suggest manufacturers time them strategically, particularly relative to the first generic approval. Once specific drug markets face generic competition, new formulations are far less common, indicating a possible diminishment of manufacturers’ ability or interest to continue incremental innovation. Another study examining new formulations based on new indications found that 32% of drugs had a new indication approved prior to generic entry, but such approvals declined afterward.^41^ The present findings of a concentration of new formulation approvals in the period leading up to first generic approval are consistent with previous research on the variety of evergreening strategies used by manufacturers to impede generic competition.^15,23,42^

New formulations have important consequences for patient access, clinical care, and health care finances. For patients, convenience or clinical benefit from a new formulation may be diminished by its expense compared with the novel drug’s generic version, which has implications for cost-related nonadherence.^43,44^ In the case of the antiepileptic drug levetiracetam, for example, the brand-name manufacturer received FDA approval for an extended-release version on September 12, 2008, before the first levetiracetam immediate-release generic version was FDA approved on November 4, 2008. A recent study^19^ found that Medicaid spent nearly $130 million between 2008 and 2016 on extended-release levetiracetam, despite therapeutic equivalence with the immediate-release version. In 2017 alone, Medicare Part D and Medicaid could have saved up to $2.6 billion by switching patients from extended-release formulations to therapeutically comparable immediate-release generics.^8^ Such examples of new formulations may have unclear patient adherence benefits^45^ and yet place financial strain on patients and the health care system.^46^ This strain may be compounded if the novel drug itself is not particularly therapeutically valuable.

This study has implications for prescription drug pricing reform, patent law, and other incentives for pharmaceutical innovation. Much attention, including in a recent letter from the FDA to the US Patent and Trademark Office^47^ and a US Department of Health and Human Services report,^48^ has been focused on the issue of strategically timed new formulations and their effect on generic competition and drug prices. The present findings highlight the importance of reforming incentives to encourage more meaningful pharmaceutical innovation. In particular, to minimize new formulations of novel drugs that do not offer therapeutic value, one approach is to align the duration of patent and regulatory exclusivities for both the novel drug and all of its new formulations with their value to patients and effect on public health.^49^ Greater duration of market exclusivity can potentially motivate subsequent innovation, but much of this innovation may be “me too” drugs that are not meaningful clinical advancements.^50^ Although we only examined therapeutic value of the novel drug in this study, the findings also underscore the need for reform of incentives for incremental innovation. For example, small changes to pharmaceutical formulations could be deemed legally insufficient to warrant new patents, or these patents could be subjected to heightened antitrust scrutiny.^51^

### Limitations

This study has limitations. First, we did not assess the potential value of new formulations, including differences in value between novel drugs and their new formulations, an important direction for future research. The research design, in which the presence of a new formulation is an outcome and not all novel drugs were followed by a new formulation, precludes an analysis of the difference between novel drugs and their new formulations.

Second, we are unable to account for the potential deterrence effect of a new formulation’s approval on the novel drug’s first generic’s approval. After a new formulation’s approval, generic manufacturers may shift their efforts to the new formulation.

Third, examining novel drugs approved through 2010 in this study meant that the minimum follow-up period of 11 years may have underestimated the number of new formulations and generics ultimately approved. However, only 6 of 206 (2.9%) novel drugs in the sample were approved in 2010. Thus, most drugs were followed for 12 or more years, which is the average duration of market exclusivity periods.^52,53^

Fourth, we only examined novel drugs’ blockbuster status, and we were unable to estimate associations with more granular degrees of drug sales. Finally, the analysis used data on regulatory drug approvals, including for generic versions, which does not necessarily indicate that the generic drug was marketed. However, this likely applies to few, if any, drugs in the sample because we excluded novel drugs with ongoing patents and exclusivity or litigation in the timing analysis.

## Conclusions

In this cross-sectional study of novel drugs approved by the FDA between 1995 and 2010, blockbuster drugs and those granted accelerated approval were more likely to be formulated again, but other measures of therapeutic value of the novel drug were not associated with new formulation approvals. Subsequent approval of a first new formulation was statistically significantly lower after the novel drug’s first generic approval. This study reinforces concerns that manufacturers are using evergreening strategies to maintain revenue and avoid generic competition. It suggests that policy makers should consider the role of new formulations more carefully to incentivize therapeutically valuable innovation and minimize strategies to avoid generic competition.

## Notes

Supplement. **eFigure 1.** Drug Sample Selection **eTable 1.** Novel Drug Therapeutic Area Classification **eFigure 2.** Proportion of Novel Tablets and Capsules Approved by the US FDA From 1995 to 2010 with New Formulation Approvals, Overall and According to Blockbuster Status, Accelerated Approval, WHO Essential Medicine, Orphan Drug Status, Innovation Status, Clinical Usefulness, Therapeutic Area, and Approval Year **eTable 2.** Measures of Therapeutic Value of Novel Tablets and Capsules Approved by the US FDA From 1995 to 2010, Including Accelerated Approval, WHO Essential Medicine, Innovation Status, and Clinical Usefulness **eMethods.** Additional New Formulation Approval Timing Analysis With Time Windows Relative to Novel Drug’s First Generic Approval **eTable 3.** Correlation Between Blockbuster Status and Measures of Therapeutic Value of Novel Tablets and Capsules Approved by the US FDA From 1995 to 2010, Including Accelerated Approval, WHO Essential Medicine, Innovation Status, and Clinical Usefulness **eTable 4.** Correlation Between Measures of Therapeutic Value of Novel Tablets and Capsules Approved by the US FDA From 1995 to 2010, Including Accelerated Approval, WHO Essential Medicine, Innovation Status, and Clinical Usefulness
