# Supplementary material for: Approvals and Timing of New Formulations of Novel Drugs Approved by the US Food and Drug Administration Between 1995 and 2010 and Followed Through 2021
Source: JAMA Health Forum. 2022 May 20;3(5):e221096. doi: 10.1001/jamahealthforum.2022.1096 (PMC9123500; doi:10.1001/jamahealthforum.2022.1096)
Supplement: Supplement. — eFigure 1. Drug Sample Selection eTable 1. Novel Drug Therapeutic Area Classification eFigure 2. Proportion of Novel Tablets and Capsules Approved by the US FDA From 1995 to 2010 with New Formulation Approvals, Overall and According to Blockbuster Status, Accelerated Approval, WHO Essential Medicine, Orphan Drug Status, Innovation Status, Clinical Usefulness, Therapeutic Area, and Approval Year eTable 2. Measures of Therapeutic Value of Novel Tablets and Capsules Approved by the US FDA From 1995 to 2010, Including Accelerated Approval, WHO Essential Medicine, Innovation Status, and Clinical Usefulness eMethods. Additional New Formulation Approval Timing Analysis With Time Windows Relative to Novel Drug’s First Generic Approval eTable 3. Correlation Between Blockbuster Status and Measures of Therapeutic Value of Novel Tablets and Capsules Approved by the US FDA From 1995 to 2010, Including Accelerated Approval, WHO Essential Medicine, Innovation Status, and Clinical Usefulness eTable 4. Correlation Between Measures of Therapeutic Value of Novel Tablets and Capsules Approved by the US FDA From 1995 to 2010, Including Accelerated Approval, WHO Essential Medicine, Innovation Status, and Clinical Usefulness [file jamahealthforum-e221096-s001.pdf]

# Supplemental Online Content

Gupta R, Morten CJ, Zhu AY, Ramachandran R, Shah ND, Ross JS. Approvals and timing of new formulations of novel drugs approved by the US Food and Drug Administration between 1995 and 2010 and followed through 2021. *JAMA Health Forum*. 2022;3(5):e221096. doi:10.1001/jamahealthforum.2022.1096

**eFigure 1.** Drug Sample Selection

**eTable 1.** Novel Drug Therapeutic Area Classification

**eFigure 2.** Proportion of Novel Tablets and Capsules Approved by the US FDA From 1995 to 2010 with New Formulation Approvals, Overall and According to Blockbuster Status, Accelerated Approval, WHO Essential Medicine, Orphan Drug Status, Innovation Status, Clinical Usefulness, Therapeutic Area, and Approval Year

**eTable 2.** Measures of Therapeutic Value of Novel Tablets and Capsules Approved by the US FDA From 1995 to 2010, Including Accelerated Approval, WHO Essential Medicine, Innovation Status, and Clinical Usefulness

**eMethods.** Additional New Formulation Approval Timing Analysis With Time Windows Relative to Novel Drug's First Generic Approval

**eTable 3.** Correlation Between Blockbuster Status and Measures of Therapeutic Value of Novel Tablets and Capsules Approved by the US FDA From 1995 to 2010, Including Accelerated Approval, WHO Essential Medicine, Innovation Status, and Clinical Usefulness

**eTable 4.** Correlation Between Measures of Therapeutic Value of Novel Tablets and Capsules Approved by the US FDA From 1995 to 2010, Including Accelerated Approval, WHO Essential Medicine, Innovation Status, and Clinical Usefulness

This supplemental material has been provided by the authors to give readers additional information about their work.

© 2022 Gupta R et al. *JAMA Health Forum.*

**eFigure 1. Drug Sample Selection**

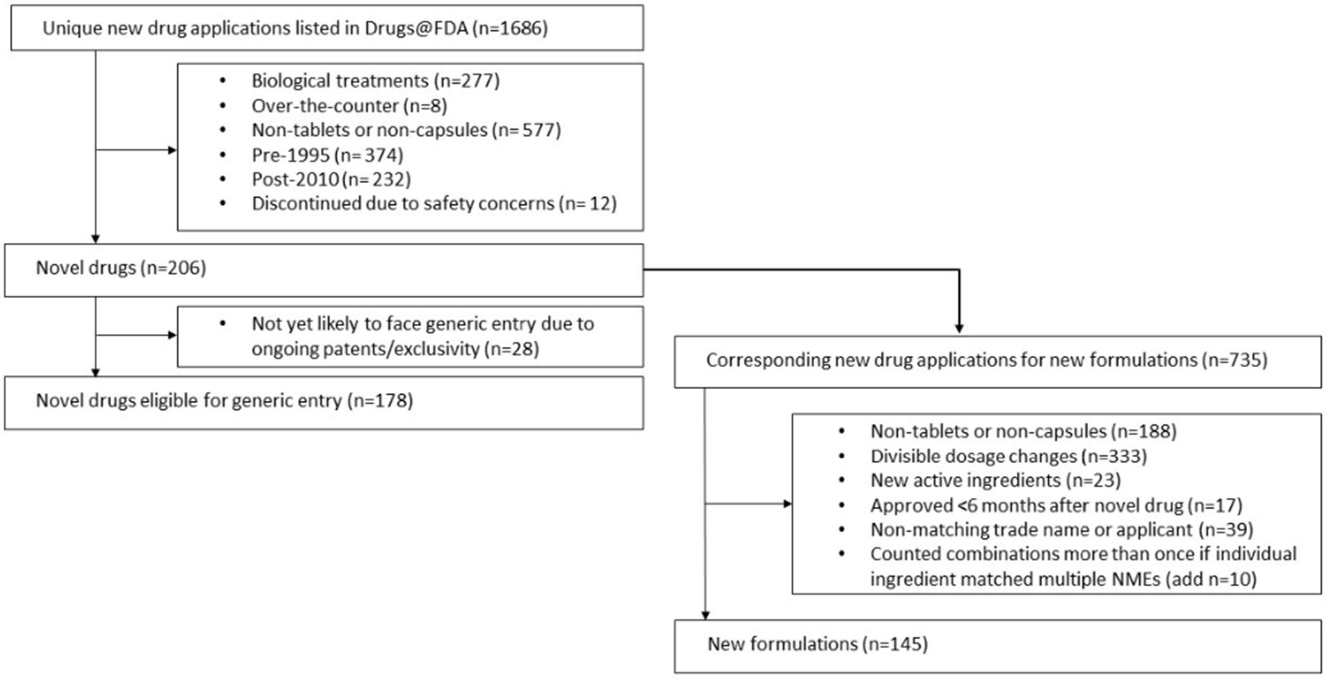


© 2022 Gupta R et al. *JAMA Health Forum.*

**eTable 1. Novel Drug Therapeutic Area Classification**

| WHO Anatomic Therapeutic Chemical Classification | Final Category |
|---|---|
| Alimentary tract and metabolism | Gastrointestinal |
| Blood and blood forming organs | Other |
| Cardiovascular system | CV, diabetes, & HLD |
| Dermatologicals | Other |
| Genitourinary system and sex hormones | Genitourinary system and sex hormones |
| Systemic hormonal preparations, excluding sex hormones and insulins | Other |
| Antiinfective for systemic use | Infectious disease |
| Antineoplastic and immunomodulating agents | Cancer |
| Musculo-skeletal system | Autoimmune and musculoskeletal |
| Nervous system | Neurology[a] |
| Antiparasitic products, insecticides, and repellants | Infectious disease |
| Respiratory system | Other |
| Sensory organs | Other |
| Various | Other |

[a]This category were subcategorized into neurology and psychiatry using WHO Anatomic Therapeutic Chemical sublevels.

© 2022 Gupta R et al. *JAMA Health Forum.*

**eFigure 2. Proportion of Novel Tablets and Capsules Approved by the US FDA From 1995 to 2010 with New Formulation Approvals, Overall and According to Blockbuster Status, Accelerated Approval, WHO Essential Medicine, Orphan Drug Status, Innovation Status, Clinical Usefulness, Therapeutic Area, and Approval Year**

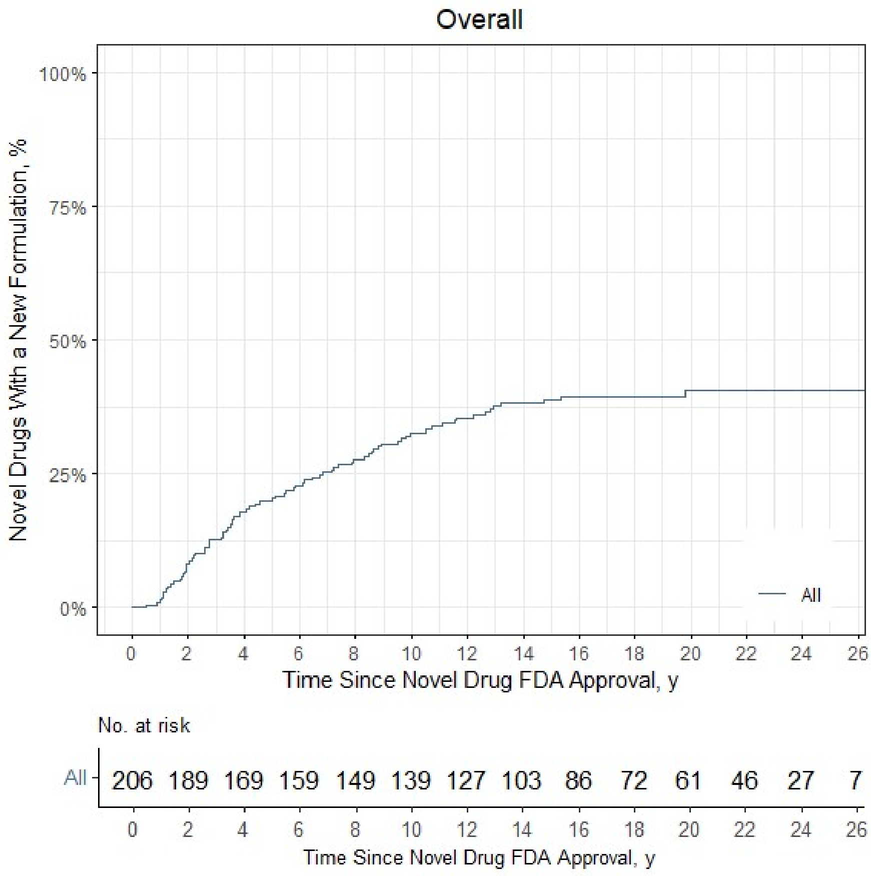


© 2022 Gupta R et al. *JAMA Health Forum.*

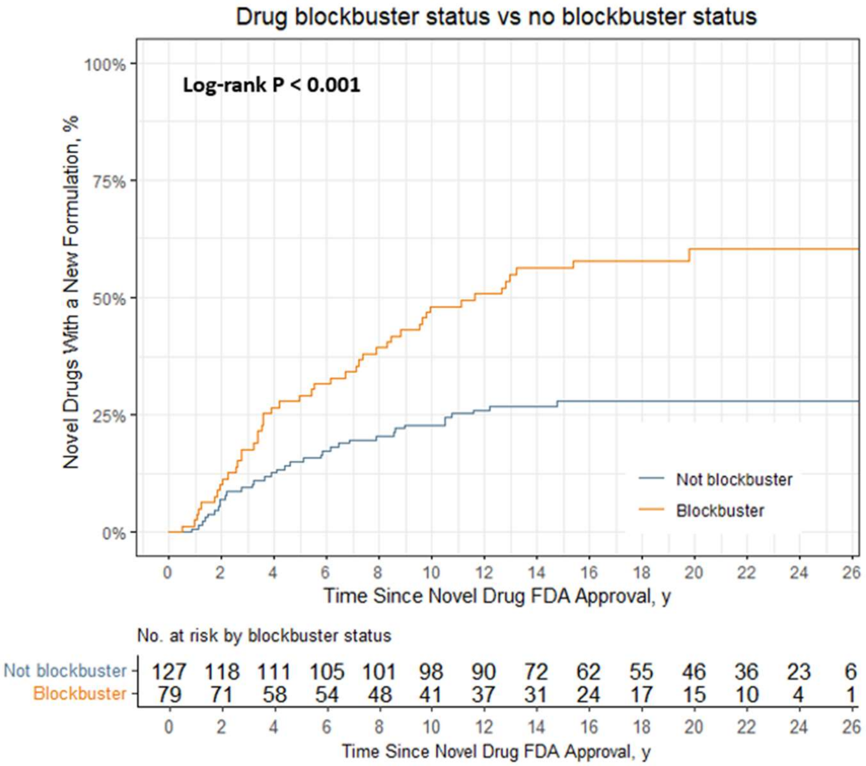


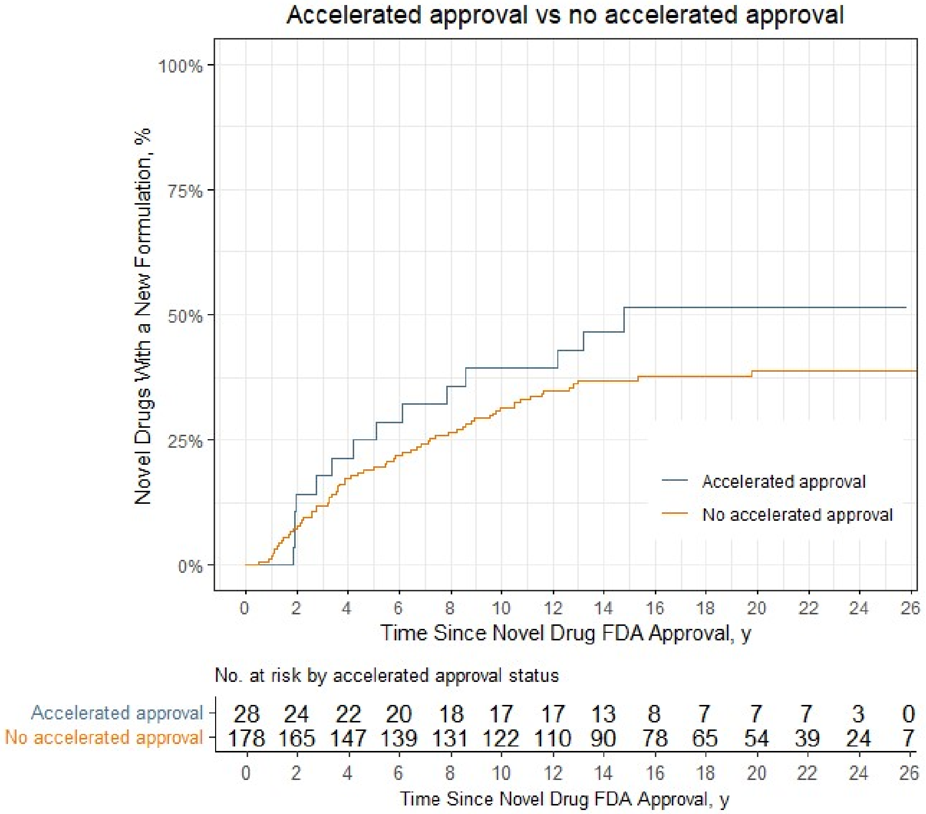


© 2022 Gupta R et al. *JAMA Health Forum.*

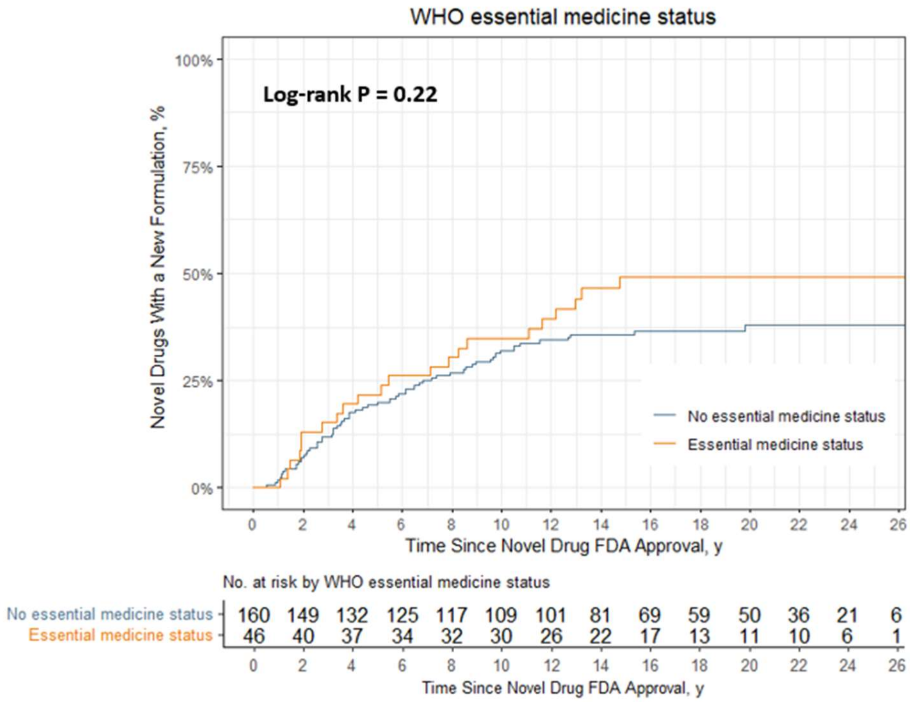


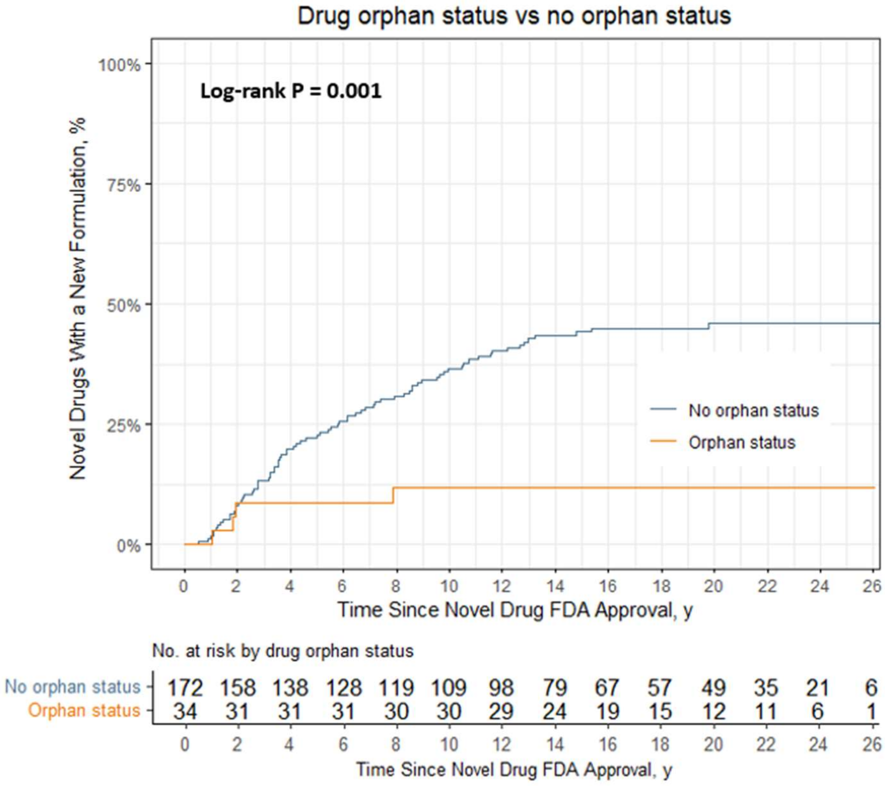


© 2022 Gupta R et al. *JAMA Health Forum.*

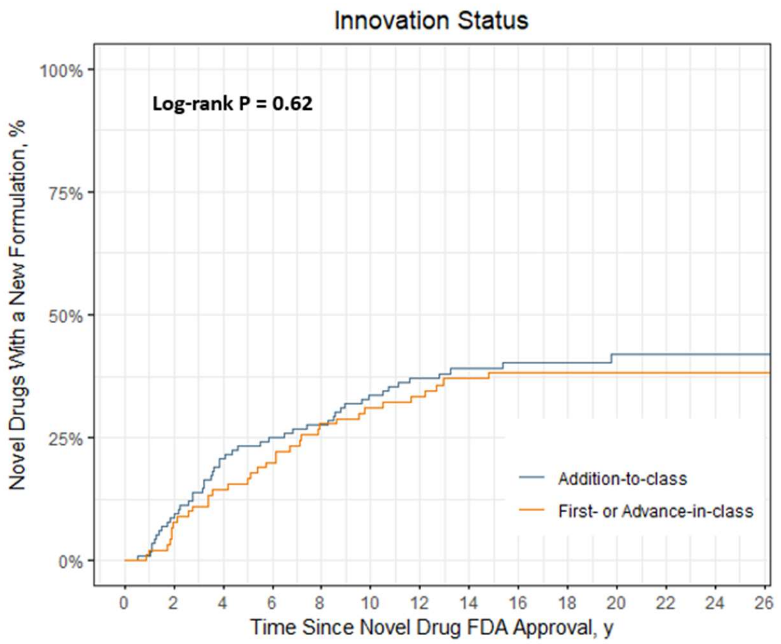


No. at risk by innovation status

| | 0 | 2 | 4 | 6 | 8 | 10 | 12 | 14 | 16 | 18 | 20 | 22 | 24 | 26 |
|---|---|---|---|---|---|---|---|---|---|---|---|---|---|---|
| Addition-to-class | 116 | 106 | 92 | 87 | 84 | 77 | 70 | 55 | 47 | 40 | 35 | 24 | 15 | 4 |
| First- or Advance-in-class | 90 | 83 | 77 | 72 | 65 | 62 | 57 | 48 | 39 | 32 | 26 | 22 | 12 | 3 |

Time Since Novel Drug FDA Approval, y

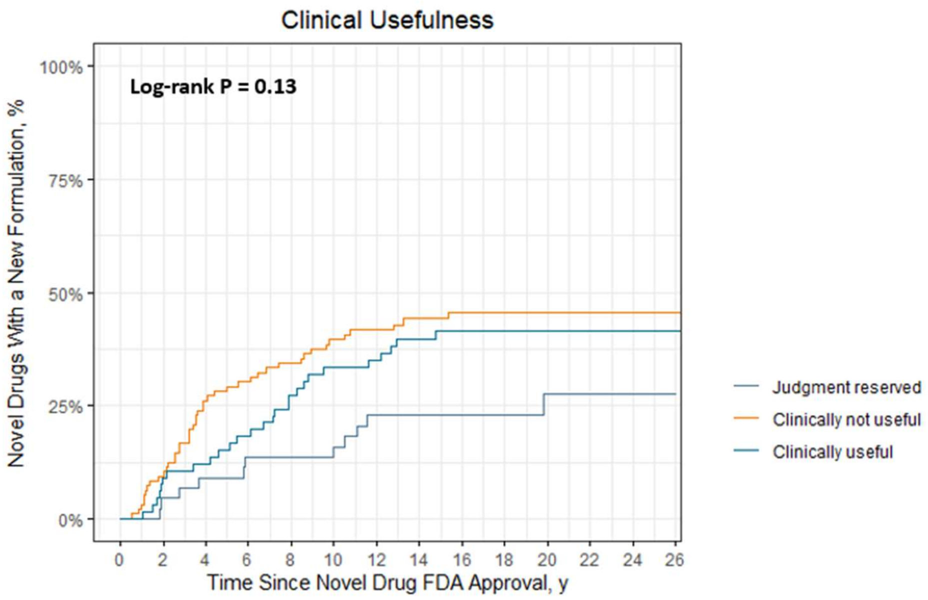


No. at risk

| | 0 | 2 | 4 | 6 | 8 | 10 | 12 | 14 | 16 | 18 | 20 | 22 | 24 | 26 |
|---|---|---|---|---|---|---|---|---|---|---|---|---|---|---|
| Judgment reserved | 44 | 42 | 40 | 38 | 38 | 37 | 32 | 26 | 22 | 17 | 15 | 13 | 7 | 1 |
| Clinically not useful | 96 | 87 | 71 | 67 | 63 | 58 | 53 | 42 | 34 | 30 | 26 | 18 | 8 | 3 |
| Clinically useful | 66 | 60 | 58 | 54 | 48 | 44 | 42 | 35 | 30 | 25 | 20 | 15 | 12 | 3 |

Time Since Novel Drug FDA Approval, y

© 2022 Gupta R et al. *JAMA Health Forum.*

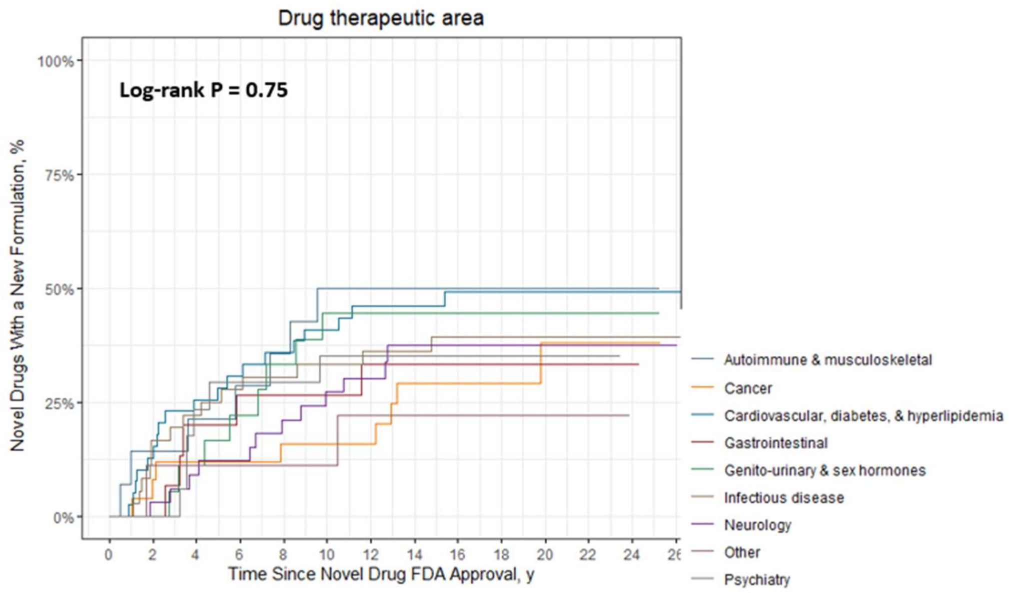


No. at risk by drug therapeutic area

| | 0 | 2 | 4 | 6 | 8 | 10 | 12 | 14 | 16 | 18 | 20 | 22 | 24 | 26 |
|---|---|---|---|---|---|---|---|---|---|---|---|---|---|---|
| Autoimmune & musculoskeletal | 14 | 12 | 11 | 10 | 9 | 7 | 7 | 6 | 6 | 6 | 4 | 3 | 2 | 0 |
| Cancer | 25 | 23 | 22 | 22 | 21 | 21 | 20 | 16 | 11 | 8 | 7 | 7 | 3 | 0 |
| Cardiovascular, diabetes, & hyperlipidemia | 39 | 34 | 29 | 27 | 25 | 23 | 21 | 18 | 15 | 15 | 14 | 11 | 9 | 4 |
| Gastrointestinal | 15 | 15 | 12 | 11 | 11 | 11 | 10 | 9 | 7 | 7 | 4 | 2 | 1 | 0 |
| Genito-urinary & sex hormones | 18 | 18 | 16 | 14 | 12 | 10 | 8 | 6 | 6 | 4 | 4 | 3 | 3 | 0 |
| Infectious disease | 36 | 30 | 28 | 26 | 25 | 24 | 23 | 21 | 17 | 13 | 12 | 10 | 7 | 2 |
| Neurology | 33 | 32 | 30 | 29 | 26 | 24 | 21 | 13 | 11 | 11 | 10 | 5 | 2 | 1 |
| Other | 9 | 8 | 8 | 8 | 8 | 8 | 6 | 5 | 5 | 3 | 2 | 2 | 0 | 0 |
| Psychiatry | 17 | 17 | 13 | 12 | 12 | 11 | 11 | 9 | 8 | 5 | 4 | 3 | 0 | 0 |

Time Since Novel Drug FDA Approval, y

© 2022 Gupta R et al. *JAMA Health Forum.*

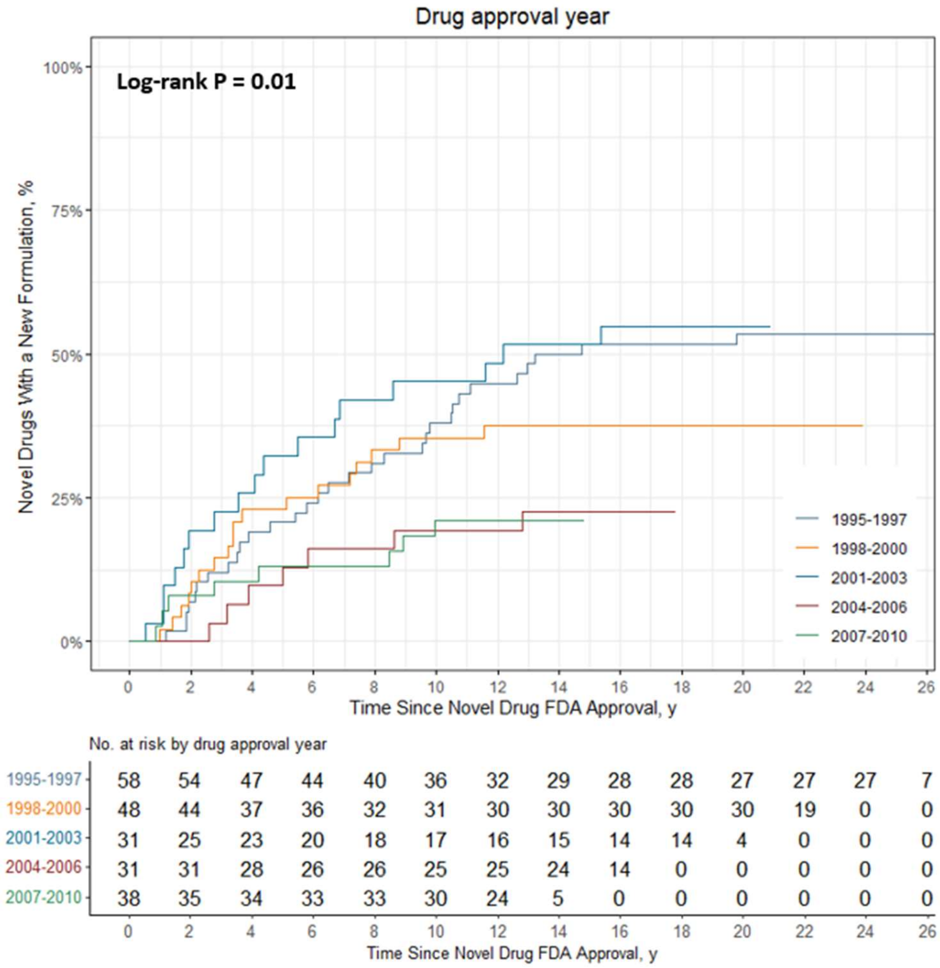


© 2022 Gupta R et al. *JAMA Health Forum.*

**eTable 2. Measures of Therapeutic Value of Novel Tablets and Capsules Approved by the US FDA From 1995 to 2010, Including Accelerated Approval, WHO Essential Medicine, Innovation Status, and Clinical Usefulness**

| Drug | Accelerated Approval | WHO Essential Medicine Listing | Innovation Status | Clinical Usefulness Based on Prescrire Rating |
|---|---|---|---|---|
| Abacavir Sulfate (Ziagen) | AA | Yes | First- or Advance-in-class | Therapeutically Helpful |
| Acamprosate Calcium (Campral) | Not AA | No | First- or Advance-in-class | Therapeutically Helpful |
| Acarbose (Precose) | Not AA | No | First- or Advance-in-class | Therapeutically Helpful |
| Adefovir Dipivoxil (Hepsera) | Not AA | No | First- or Advance-in-class | Therapeutically Helpful |
| Albendazole (Albenza) | Not AA | Yes | First- or Advance-in-class | Therapeutically Helpful |
| Alendronate Sodium (Fosamax) | Not AA | No | First- or Advance-in-class | Therapeutically Helpful |
| Alfuzosin Hydrochloride (Uroxatral) | Not AA | No | Addition-to-class | Therapeutically Helpful |
| Aliskiren Hemifumarate (Tekturna) | Not AA | No | First- or Advance-in-class | Not helpful |
| Almotriptan Malate (Axert) | Not AA | No | Addition-to-class | Not helpful |
| Alosetron Hydrochloride (Lotronex) | Not AA | No | First- or Advance-in-class | Not helpful |
| Alvimopan (Entereg) | Not AA | No | Addition-to-class | Judgment Reserved |
| Ambrisentan (Letairis) | Not AA | No | Addition-to-class | Not helpful |
| Amprenavir (Agenerase) | AA | No | Addition-to-class | Not helpful |
| Anagrelide Hydrochloride (Agrylin) | Not AA | No | First- or Advance-in-class | Judgment Reserved |
| Anastrozole (Arimidex) | Not AA | Yes | First- or Advance-in-class | Therapeutically Helpful |
| Aprepitant (Emend) | Not AA | Yes | First- or Advance-in-class | Not helpful |
| Aripiprazole (Abilify) | Not AA | No | First- or Advance-in-class | Not helpful |
| Artemether; Lumefantrine (Coartem) | Not AA | Yes | First- or Advance-in-class | Therapeutically Helpful |
| Asenapine Maleate (Saphris) | Not AA | No | Addition-to-class | Not helpful |
| Atazanavir Sulfate (Reyataz) | Not AA | Yes | First- or Advance-in-class | Therapeutically Helpful |
| Atomoxetine Hydrochloride (Strattera) | Not AA | No | First- or Advance-in-class | Not helpful |
| Atorvastatin Calcium (Lipitor) | Not AA | Yes | First- or Advance-in-class | Therapeutically Helpful |
| Balsalazide Disodium (Colazal) | Not AA | No | Addition-to-class | Judgment Reserved |
| Bexarotene (Targretin) | Not AA | No | First- or Advance-in-class | Not helpful |
| Bicalutamide (Casodex) | AA | Yes | Addition-to-class | Not helpful |

© 2022 Gupta R et al. *JAMA Health Forum.*

| | | | | |
|---|---|---|---|---|
| Bismuth Subcitrate Potassium; Metronidazole; Tetracycline Hydrochloride (Pylera) | Not AA | No | Addition-to-class | Not helpful |
| Bosentan (Tracleer) | Not AA | No | First- or Advance-in-class | Therapeutically Helpful |
| Cabergoline (Dostinex) | Not AA | No | Addition-to-class | Therapeutically Helpful |
| Candesartan Cilexetil (Atacand) | Not AA | No | Addition-to-class | Not helpful |
| Capecitabine (Xeloda) | AA | Yes | First- or Advance-in-class | Not helpful |
| Carvedilol (Coreg) | Not AA | Yes | Addition-to-class | Judgment Reserved |
| Cefdinir (Omnicef) | Not AA | No | Addition-to-class | Judgment Reserved |
| Ceftibuten Dihydrate (Cedax) | Not AA | No | Addition-to-class | Judgment Reserved |
| Celecoxib (Celebrex) | Not AA | No | First- or Advance-in-class | Not helpful |
| Cetirizine Hydrochloride (Zyrtec Allergy) | Not AA | Yes | Addition-to-class | Therapeutically Helpful |
| Cevimeline Hydrochloride (Evoxac) | Not AA | No | Addition-to-class | Judgment Reserved |
| Cilostazol (Pletal) | Not AA | No | First- or Advance-in-class | Therapeutically Helpful |
| Cinacalcet Hydrochloride (Sensipar) | Not AA | No | First- or Advance-in-class | Judgment Reserved |
| Citalopram Hydrobromide (Celexa) | Not AA | Yes | Addition-to-class | Not helpful |
| Clopidogrel Bisulfate (Plavix) | Not AA | Yes | First- or Advance-in-class | Therapeutically Helpful |
| Colesevelam Hydrochloride (Welchol) | Not AA | No | Addition-to-class | Not helpful |
| Dabigatran Etexilate Mesylate (Pradaxa) | Not AA | Yes | First- or Advance-in-class | Therapeutically Helpful |
| Dalfampridine (Ampyra) | Not AA | No | First- or Advance-in-class | Judgment Reserved |
| Darifenacin Hydrobromide (Enablex) | Not AA | No | Addition-to-class | Judgment Reserved |
| Darunavir (Prezista) | AA | Yes | First- or Advance-in-class | Therapeutically Helpful |
| Dasatinib (Sprycel) | AA | Yes | First- or Advance-in-class | Therapeutically Helpful |
| Delavirdine Mesylate (Rescriptor) | AA | No | First- or Advance-in-class | Judgment Reserved |
| Desloratadine (Clarinex) | Not AA | No | Addition-to-class | Not helpful |
| Desvenlafaxine Succinate (Pristiq) | Not AA | No | Addition-to-class | Not helpful |
| Dienogest; Estradiol Valerate (Natazia) | Not AA | No | Addition-to-class | Not helpful |
| Dirithromycin (Dynabac) | Not AA | No | Addition-to-class | Not helpful |
| Dofetilide (Tikosyn) | Not AA | No | Addition-to-class | Judgment Reserved |
| Dolasetron Mesylate (Anzemet) | Not AA | Yes | Addition-to-class | Judgment Reserved |
| Donepezil Hydrochloride (Aricept) | Not AA | No | First- or Advance-in-class | Therapeutically Helpful |
| Doxercalciferol (Hectorol) | Not AA | No | Addition-to-class | Judgment Reserved |

© 2022 Gupta R et al. *JAMA Health Forum.*

| | | | | |
|---|---|---|---|---|
| Dronedarone Hydrochloride (Multaq) | Not AA | No | First- or Advance-in-class | Therapeutically Helpful |
| Drospirenone; Ethinyl Estradiol (Yasmin) | Not AA | No | Addition-to-class | Not helpful |
| Duloxetine Hydrochloride (Cymbalta) | Not AA | No | Addition-to-class | Not helpful |
| Dutasteride (Avodart) | Not AA | No | Addition-to-class | Not helpful |
| Efavirenz (Sustiva) | AA | Yes | First- or Advance-in-class | Therapeutically Helpful |
| Eletriptan Hydrobromide (Relpax) | Not AA | No | Addition-to-class | Not helpful |
| Eltrombopag Olamine (Promacta) | AA | No | First- or Advance-in-class | Judgment Reserved |
| Emtricitabine (Emtriva) | Not AA | No | Addition-to-class | Not helpful |
| Entacapone (Comtan) | Not AA | No | Addition-to-class | Judgment Reserved |
| Entecavir (Baraclude) | Not AA | Yes | First- or Advance-in-class | Therapeutically Helpful |
| Eplerenone (Inspra) | Not AA | No | Addition-to-class | Therapeutically Helpful |
| Eprosartan Mesylate (Teveten) | Not AA | No | Addition-to-class | Not helpful |
| Erlotinib Hydrochloride (Tarceva) | Not AA | Yes | First- or Advance-in-class | Not helpful |
| Eszopiclone (Lunesta) | Not AA | No | Addition-to-class | Judgment Reserved |
| Etravirine (Intelence) | AA | No | First- or Advance-in-class | Therapeutically Helpful |
| Everolimus (Afinitor) | Not AA | Yes | Addition-to-class | Therapeutically Helpful |
| Exemestane (Aromasin) | Not AA | No | Addition-to-class | Not helpful |
| Ezetimibe (Zetia) | Not AA | No | First- or Advance-in-class | Not helpful |
| Febuxostat (Uloric) | Not AA | No | Addition-to-class | Not helpful |
| Ferric Hexacyanoferrate(Ii) (Radiogardase (Prussian Blue)) | Not AA | Yes | First- or Advance-in-class | Judgment Reserved |
| Fesoterodine Fumarate (Toviaz) | Not AA | No | Addition-to-class | Judgment Reserved |
| Fexofenadine Hydrochloride (Allegra) | Not AA | Yes | Addition-to-class | Not helpful |
| Fingolimod Hydrochloride (Gilenya) | Not AA | No | First- or Advance-in-class | Not helpful |
| Frovatriptan Succinate (Frova) | Not AA | No | Addition-to-class | Not helpful |
| Galantamine Hydrobromide (Razadyne) | Not AA | No | Addition-to-class | Not helpful |
| Gefitinib (Iressa) | AA | Yes | First- or Advance-in-class | Therapeutically Helpful |
| Glimepiride (Amaryl) | Not AA | No | Addition-to-class | Not helpful |
| Ibandronate Sodium (Boniva) | Not AA | No | Addition-to-class | Not helpful |
| Iloperidone (Fanapt) | Not AA | No | Addition-to-class | Judgment Reserved |
| Imatinib Mesylate (Gleevec) | AA | Yes | First- or Advance-in-class | Therapeutically Helpful |
| Indinavir Sulfate (Crixivan) | AA | No | First- or Advance-in-class | Therapeutically Helpful |
| Irbesartan (Avapro) | Not AA | No | Addition-to-class | Therapeutically Helpful |

© 2022 Gupta R et al. *JAMA Health Forum.*

| | | | | |
|---|---|---|---|---|
| Ivermectin (Stromectol) | Not AA | Yes | First- or Advance-in-class | Therapeutically Helpful |
| Lacosamide (Vimpat) | Not AA | No | Addition-to-class | Not helpful |
| Lamivudine (Epivir) | AA | Yes | First- or Advance-in-class | Judgment Reserved |
| Lansoprazole (Prevacid) | Not AA | No | Addition-to-class | Not helpful |
| Lanthanum Carbonate (Fosrenol) | Not AA | No | Addition-to-class | Not helpful |
| Lapatinib Ditosylate (Tykerb) | Not AA | No | Addition-to-class | Therapeutically Helpful |
| Leflunomide (Arava) | Not AA | No | First- or Advance-in-class | Not helpful |
| Lenalidomide (Revlimid) | Not AA | Yes | First- or Advance-in-class | Judgment Reserved |
| Letrozole (Femara) | Not AA | No | Addition-to-class | Judgment Reserved |
| Levetiracetam (Keppra) | Not AA | No | Addition-to-class | Therapeutically Helpful |
| Linezolid (Zyvox) | Not AA | Yes | First- or Advance-in-class | Therapeutically Helpful |
| Lisdexamfetamine Dimesylate (Vyvanse) | Not AA | No | Addition-to-class | Judgment Reserved |
| Lopinavir; Ritonavir (Kaletra) | AA | Yes | First- or Advance-in-class | Therapeutically Helpful |
| Losartan Potassium (Cozaar) | Not AA | Yes | First- or Advance-in-class | Therapeutically Helpful |
| Lubiprostone (Amitiza) | Not AA | No | First- or Advance-in-class | Judgment Reserved |
| Lurasidone Hydrochloride (Latuda) | Not AA | No | Addition-to-class | Judgment Reserved |
| Maraviroc (Selzentry) | AA | No | First- or Advance-in-class | Judgment Reserved |
| Meloxicam (Mobic) | Not AA | No | Addition-to-class | Not helpful |
| Memantine Hydrochloride (Namenda) | Not AA | No | First- or Advance-in-class | Therapeutically Helpful |
| Metformin Hydrochloride (Glucophage) | Not AA | Yes | First- or Advance-in-class | Therapeutically Helpful |
| Midodrine Hydrochloride (Proamatine) | AA | No | First- or Advance-in-class | Therapeutically Helpful |
| Mifepristone (Mifeprex) | Not AA | No | First- or Advance-in-class | Therapeutically Helpful |
| Miglitol (Glyset) | Not AA | No | Addition-to-class | Therapeutically Helpful |
| Miglustat (Zavesca) | Not AA | No | First- or Advance-in-class | Therapeutically Helpful |
| Milnacipran Hydrochloride (Savella) | Not AA | No | Addition-to-class | Not helpful |
| Mirtazapine (Remeron) | Not AA | No | Addition-to-class | Therapeutically Helpful |
| Modafinil (Provigil) | Not AA | No | First- or Advance-in-class | Therapeutically Helpful |
| Moexipril Hydrochloride (Univasc) | Not AA | No | Addition-to-class | Not helpful |
| Montelukast Sodium (Singulair) | Not AA | No | Addition-to-class | Judgment Reserved |
| Moxifloxacin Hydrochloride (Avelox) | Not AA | Yes | Addition-to-class | Not helpful |

© 2022 Gupta R et al. *JAMA Health Forum.*

| | | | | |
|---|---|---|---|---|
| Mycophenolate Mofetil (Cellcept) | Not AA | No | First- or Advance-in-class | Therapeutically Helpful |
| Naratriptan Hydrochloride (Amerge) | Not AA | No | Addition-to-class | Therapeutically Helpful |
| Nateglinide (Starlix) | Not AA | No | Addition-to-class | Not helpful |
| Nebivolol Hydrochloride (Bystolic) | Not AA | No | Addition-to-class | Not helpful |
| Nelfinavir Mesylate (Viracept) | AA | No | First- or Advance-in-class | Therapeutically Helpful |
| Nevirapine (Viramune) | AA | Yes | First- or Advance-in-class | Therapeutically Helpful |
| Nilotinib Hydrochloride (Tasigna) | AA | Yes | Addition-to-class | Judgment Reserved |
| Nilutamide (Nilandron) | Not AA | Yes | Addition-to-class | Not helpful |
| Nisoldipine (Sular) | Not AA | No | Addition-to-class | Not helpful |
| Nitisinone (Orfadin) | Not AA | No | First- or Advance-in-class | Therapeutically Helpful |
| Olanzapine (Zyprexa) | Not AA | No | Addition-to-class | Not helpful |
| Olmesartan Medoxomil (Benicar) | Not AA | No | Addition-to-class | Not helpful |
| Omega-3-Acid Ethyl Esters (Lovaza) | Not AA | No | First- or Advance-in-class | Not helpful |
| Orlistat (Xenical) | Not AA | No | First- or Advance-in-class | Not helpful |
| Oseltamivir Phosphate (Tamiflu) | Not AA | Yes | First- or Advance-in-class | Not helpful |
| Oxcarbazepine (Trileptal) | Not AA | No | Addition-to-class | Therapeutically Helpful |
| Paliperidone (Invega) | Not AA | Yes | Addition-to-class | Not helpful |
| Pantoprazole Sodium (Protonix) | Not AA | No | Addition-to-class | Therapeutically Helpful |
| Pazopanib Hydrochloride (Votrient) | Not AA | No | Addition-to-class | Not helpful |
| Pentosan Polysulfate Sodium (Elmiron) | Not AA | No | First- or Advance-in-class | Not helpful |
| Pioglitazone Hydrochloride (Actos) | Not AA | No | First- or Advance-in-class | Not helpful |
| Pitavastatin Calcium (Livalo) | Not AA | No | Addition-to-class | Not helpful |
| Pramipexole Dihydrochloride (Mirapex) | Not AA | No | First- or Advance-in-class | Therapeutically Helpful |
| Prasugrel Hydrochloride (Effient) | Not AA | No | First- or Advance-in-class | Not helpful |
| Pregabalin (Lyrica) | Not AA | No | Addition-to-class | Not helpful |
| Quetiapine Fumarate (Seroquel) | Not AA | No | Addition-to-class | Not helpful |
| Rabeprazole Sodium (Aciphex) | Not AA | No | Addition-to-class | Not helpful |
| Raloxifene Hydrochloride (Evista) | Not AA | No | First- or Advance-in-class | Not helpful |
| Raltegravir Potassium (Isentress) | AA | Yes | First- or Advance-in-class | Therapeutically Helpful |
| Ramelteon (Rozerem) | Not AA | No | First- or Advance-in-class | Not helpful |
| Ranolazine (Ranexa) | Not AA | No | First- or Advance-in-class | Not helpful |
| Rasagiline Mesylate (Azilect) | Not AA | No | Addition-to-class | Not helpful |

© 2022 Gupta R et al. *JAMA Health Forum.*

| | | | | |
|---|---|---|---|---|
| Repaglinide (Prandin) | Not AA | No | First- or Advance-in-class | Not helpful |
| Rifapentine (Priftin) | AA | Yes | First- or Advance-in-class | Judgment Reserved |
| Rifaximin (Xifaxan) | Not AA | No | Addition-to-class | Judgment Reserved |
| Riluzole (Rilutek) | Not AA | No | First- or Advance-in-class | Therapeutically Helpful |
| Risedronate Sodium (Actonel) | Not AA | No | Addition-to-class | Not helpful |
| Rivastigmine Tartrate (Exelon) | Not AA | No | Addition-to-class | Not helpful |
| Rizatriptan Benzoate (Maxalt) | Not AA | No | Addition-to-class | Judgment Reserved |
| Ropinirole Hydrochloride (Requip) | Not AA | No | Addition-to-class | Not helpful |
| Rosiglitazone Maleate (Avandia) | Not AA | No | First- or Advance-in-class | Not helpful |
| Rosuvastatin Calcium (Crestor) | Not AA | No | Addition-to-class | Not helpful |
| Rufinamide (Banzel) | Not AA | No | Addition-to-class | Judgment Reserved |
| Sapropterin Dihydrochloride (Kuvan) | Not AA | No | First- or Advance-in-class | Therapeutically Helpful |
| Saquinavir Mesylate (Invirase) | AA | No | First- or Advance-in-class | Judgment Reserved |
| Saxagliptin Hydrochloride (Onglyza) | Not AA | No | Addition-to-class | Not helpful |
| Sevelamer Hydrochloride (Renagel) | Not AA | No | Addition-to-class | Therapeutically Helpful |
| Sildenafil Citrate (Viagra) | Not AA | No | First- or Advance-in-class | Therapeutically Helpful |
| Silodosin (Rapaflo) | Not AA | No | Addition-to-class | Not helpful |
| Sitagliptin Phosphate (Januvia) | Not AA | No | First- or Advance-in-class | Not helpful |
| Solifenacin Succinate (Vesicare) | Not AA | No | Addition-to-class | Judgment Reserved |
| Sorafenib Tosylate (Nexavar) | Not AA | No | First- or Advance-in-class | Therapeutically Helpful |
| Sunitinib Malate (Sutent) | AA | No | First- or Advance-in-class | Therapeutically Helpful |
| Tadalafil (Cialis) | Not AA | No | Addition-to-class | Not helpful |
| Tamsulosin Hydrochloride (Flomax) | Not AA | No | Addition-to-class | Not helpful |
| Tapentadol Hydrochloride (Nucynta) | Not AA | No | Addition-to-class | Judgment Reserved |
| Telbivudine (Tyzeka) | Not AA | No | Addition-to-class | Not helpful |
| Telithromycin (Ketek) | Not AA | No | Addition-to-class | Not helpful |
| Telmisartan (Micardis) | Not AA | No | Addition-to-class | Not helpful |
| Temozolomide (Temodar) | AA | No | First- or Advance-in-class | Not helpful |
| Tenofovir Disoproxil Fumarate (Viread) | AA | Yes | First- or Advance-in-class | Not helpful |
| Tetrabenazine (Xenazine) | Not AA | No | First- or Advance-in-class | Judgment Reserved |
| Thalidomide (Thalomid) | AA | Yes | First- or Advance-in-class | Therapeutically Helpful |

© 2022 Gupta R et al. *JAMA Health Forum.*

| | | | | |
|---|---|---|---|---|
| Tiagabine Hydrochloride (Gabitril) | Not AA | No | Addition-to-class | Therapeutically Helpful |
| Tiludronate Disodium (Skelid) | Not AA | No | Addition-to-class | Judgment Reserved |
| Tinidazole (Tindamax) | Not AA | Yes | Addition-to-class | Therapeutically Helpful |
| Tipranavir (Aptivus) | AA | No | Addition-to-class | Therapeutically Helpful |
| Tizanidine Hydrochloride (Zanaflex) | Not AA | No | First- or Advance-in-class | Judgment Reserved |
| Tolcapone (Tasmar) | Not AA | No | First- or Advance-in-class | Not helpful |
| Tolterodine Tartrate (Detrol) | Not AA | No | Addition-to-class | Not helpful |
| Tolvaptan (Samsca) | Not AA | No | Addition-to-class | Not helpful |
| Topiramate (Topamax) | Not AA | No | Addition-to-class | Therapeutically Helpful |
| Toremifene Citrate (Fareston) | Not AA | No | Addition-to-class | Not helpful |
| Tramadol Hydrochloride (Ultram) | Not AA | No | Addition-to-class | Not helpful |
| Trandolapril (Mavik) | Not AA | No | Addition-to-class | Not helpful |
| Trospium Chloride (Sanctura) | Not AA | No | Addition-to-class | Not helpful |
| Ulipristal Acetate (Ella) | Not AA | Yes | Addition-to-class | Not helpful |
| Valsartan (Diovan) | Not AA | No | Addition-to-class | Not helpful |
| Vardenafil Hydrochloride (Levitra) | Not AA | No | Addition-to-class | Not helpful |
| Varenicline Tartrate (Chantix) | Not AA | Yes | First- or Advance-in-class | Not helpful |
| Vigabatrin (Sabril) | Not AA | No | Addition-to-class | Therapeutically Helpful |
| Voriconazole (Vfend) | Not AA | Yes | Addition-to-class | Therapeutically Helpful |
| Vorinostat (Zolinza) | Not AA | No | First- or Advance-in-class | Judgment Reserved |
| Zafirlukast (Accolate) | Not AA | No | First- or Advance-in-class | Judgment Reserved |
| Zaleplon (Sonata) | Not AA | No | Addition-to-class | Judgment Reserved |
| Zileuton (Zyflo) | Not AA | No | Addition-to-class | Judgment Reserved |
| Ziprasidone Hydrochloride (Geodon) | Not AA | No | Addition-to-class | Judgment Reserved |
| Zolmitriptan (Zomig) | Not AA | No | Addition-to-class | Not helpful |
| Zonisamide (Zonegran) | Not AA | No | Addition-to-class | Not helpful |

[a] Categorization was based on Prescrire ratings. Drugs that were categorized by Prescrire as “Possibly Helpful,” “Offers an Advantage,” “A Real Advance,” or “Bravo,” were grouped together as “Clinically Useful.” Drugs that were categorized as “Nothing New” and “Not Acceptable” were grouped together as “Clinically Not Useful.” The remaining drugs were categorized as “Judgment Reserved.”

© 2022 Gupta R et al. *JAMA Health Forum.*

**eMethods. Additional New Formulation Approval Timing Analysis With Time Windows Relative to Novel Drug's First Generic Approval**

We conducted an additional timing analysis to account for regulatory delays in approval by examining the likelihood of a new formulation in 4 time windows: greater than 6 years before, 2 to 6 years before, 2 years before and after, and greater than 2 years after the novel drug's first generic approval. For the time window around the generic approval, the 2-year period prior to generic approval accounts for the potential time from a generic manufacturer's application submission to FDA approval; the 2-year period after the generic's approval accounts for the possibility of regulatory delays in new formulation approvals, particularly if the company unsuccessfully attempted to time the new formulation approval with the first generic approval. We then performed multinomial logistic regression analysis with presence of the first new formulation in each of the 4 time periods relative to the novel drug's first generic version's approval timing.

**Results:**

Among the 65 novel drugs with both at least 1 new formulation and a generic, 32 (49%) new formulations were approved more than 6 years before the novel drugs' first generic approval, 14 (22%) were approved 2 to 6 years before generic approval, 15 (23%) were approved 2 years before and after generic approval, and 4 (6%) were approved more than 2 years after generic approval. First new formulations were less likely to be approved in the time period 2 to 6 years before the novel drug's first generic approval (RRR=0.44, 95% CI, 0.23-0.82; *P*=0.01) and 2 years before and after generic approval (RRR=0.47, 95% CI, 0.25-0.87; *P*=0.015), as compared to more than 6 years prior to first generic approval. First new formulations were significantly less likely to be approved more than 2 years after the first generic approval (RRR=0.13, 95% CI, 0.04-0.35; *P*<0.001).

**eTable 3. Correlation Between Blockbuster Status and Measures of Therapeutic Value of Novel Tablets and Capsules Approved by the US FDA From 1995 to 2010, Including Accelerated Approval, WHO Essential Medicine, Innovation Status, and Clinical Usefulness**

| **Measure of Therapeutic Value** | **Blockbuster status** | ***P* value** |
|---|---|---|
| Accelerated approval | 0.10 | 0.76 |
| WHO essential medicine | 8.27 | 0.004 |
| Innovation status | 1.68 | 0.20 |
| Clinical Usefulness Based on Prescrire Rating | 4.27 | 0.12 |

Pearson-$\chi^2$

© 2022 Gupta R et al. *JAMA Health Forum.*

**eTable 4. Correlation Between Measures of Therapeutic Value of Novel Tablets and Capsules Approved by the US FDA From 1995 to 2010, Including Accelerated Approval, WHO Essential Medicine, Innovation Status, and Clinical Usefulness**

| | **WHO Essential Medicine Listing** | ***P* value** | **Innovation Status** | ***P* value** | **Clinical Usefulness Based on Prescrire Rating** | ***P* value** |
|---|---|---|---|---|---|---|
| Accelerated approval | 22.64 | <0.001 | 23.26 | <0.001 | 12.32 | 0.002 |
| WHO essential medicine | - | - | 16.12 | <0.001 | 16.40 | <0.001 |
| Innovation status | - | - | - | - | 27.34 | <0.001 |

Pearson-$\chi^2$

© 2022 Gupta R et al. *JAMA Health Forum.*
